# Supplementary material for: Molecular dynamics simulations reveal the selectivity mechanism of structurally similar agonists to TLR7 and TLR8
Source: PLoS One. 2022 Apr 22;17(4):e0260565. doi: 10.1371/journal.pone.0260565 (PMC9032342; doi:10.1371/journal.pone.0260565)
Supplement: S1 File — Details of hydrogen bonds between agonists and TLR7 and TLR8. The binding free energies between each residue of TLR7 and TLR8 and agonists. (DOCX) [file pone.0260565.s001.docx]

**Molecular dynamics simulations reveal the selectivity mechanism of structurally similar agonists to TLR7 and TLR8**

Xiaoyu Wang^1^, Yu Chen^1^, Steven Zhang^2^, Jinxia Nancy Deng^3^*

1 Computational Chemistry Department, Shanghai ChemPartner Co., Ltd., 576 Libing Road, Shanghai 201203, China

2 Chemistry Department, Shanghai ChemPartner Co., Ltd., 576 Libing Road, Shanghai 201203, China

3* corresponding author, Computational Chemistry Department, ChemPartner, 280 Utah Ave, South San Francisco, CA, 94080

**Table S1. Energy decomposition of binding free energy of H to TLR7.** All units are kcal/mol.

| **TLR7-H** Binding free energy: -50.50 kcal/mol | | | | | |
| --- | --- | --- | --- | --- | --- |
| **Residue** | **ΔE_vdW_** | **ΔE_elec_** | **ΔE_polar_** | **ΔE_surf_** | **Total** |
| **A-F351** | -2.163 | 0.125 | -1.300 | -0.166 | -3.503 |
| **A-Y356** | -1.172 | -0.629 | 0.445 | -0.188 | -1.544 |
| **A-V381** | -1.450 | -0.630 | 1.202 | -0.176 | -1.054 |
| **A-F408** | -3.533 | -0.177 | -0.017 | -0.273 | -4.001 |
| **B-D555** | 0.858 | -44.538 | 36.959 | -0.047 | -6.768 |
| **B-L557** | -2.599 | 1.539 | -1.115 | -0.335 | -2.511 |
| **B-I585** | -1.992 | 0.741 | 0.038 | -0.068 | -1.281 |
| **B-T586** | -1.924 | -5.717 | 3.100 | -0.098 | -4.639 |

**Table S2. Energy decomposition of binding free energy of R to TLR7.** All units are kcal/mol.

| **TLR7-R** Binding free energy: -44.21 kcal/mol | | | | | |
| --- | --- | --- | --- | --- | --- |
| **Residue** | **ΔE_vdW_** | **ΔE_elec_** | **ΔE_polar_** | **ΔE_surf_** | **Total** |
| **A-F351** | -2.020 | 0.454 | -1.131 | -0.152 | -2.849 |
| **A-Y356** | -1.199 | -0.392 | 0.288 | -0.181 | -1.484 |
| **A-F408** | -4.020 | -0.655 | 0.263 | -0.288 | -4.699 |
| **B-D555** | 0.498 | -46.895 | 41.939 | -0.084 | -4.542 |
| **B-L557** | -2.406 | 1.320 | -0.704 | -0.359 | -2.149 |
| **B-I585** | -1.847 | 0.863 | -0.396 | -0.056 | -1.436 |
| **B-T586** | -1.856 | -5.950 | 2.842 | -0.144 | -5.109 |

**Table S3. Energy decomposition of binding free energy of G to TLR7.** All units are kcal/mol.

| **TLR7-G** Binding free energy: -33.65 kcal/mol | | | | | |
| --- | --- | --- | --- | --- | --- |
| **Residue** | **ΔE_vdW_** | **ΔE_elec_** | **ΔE_polar_** | **ΔE_surf_** | **Total** |
| **A-F351** | -0.634 | 0.595 | -0.834 | -0.156 | -1.028 |
| **A-Y356** | -1.270 | -0.199 | 0.092 | -0.212 | -1.590 |
| **A-F408** | -3.639 | -1.243 | 0.366 | -0.318 | -4.834 |
| **B-D555** | 1.159 | -68.122 | 57.091 | -0.037 | -9.909 |
| **B-L557** | -2.623 | 1.106 | -0.228 | -0.387 | -2.132 |
| **B-T586** | -1.595 | -7.604 | 3.923 | -0.151 | -5.427 |

**Table S4 Energy decomposition of binding free energy of H to TLR8.** All units are kcal/mol.

| **TLR8-H** Binding free energy: -42.09 kcal/mol | | | | | |
| --- | --- | --- | --- | --- | --- |
| **Residue** | **ΔE_vdW_** | **ΔE_elec_** | **ΔE_polar_** | **ΔE_surf_** | **Total** |
| **A-Y348** | -1.954 | -0.046 | 0.039 | -0.162 | -2.123 |
| **A-Y353** | -1.173 | -0.827 | 0.788 | -0.192 | -1.405 |
| **A-F405** | -3.417 | -0.416 | 0.096 | -0.292 | -4.029 |
| **B-D543** | 0.553 | -46.112 | 40.805 | -0.061 | -4.814 |
| **B-V573** | -1.857 | 1.453 | -0.701 | -0.085 | -1.190 |
| **B-T574** | -1.809 | -6.095 | 3.702 | -0.149 | -4.351 |

**Table S5. Energy decomposition of binding free energy of R to TLR8.** All units are kcal/mol.

| **TLR8-R** Binding free energy: -39.55kcal/mol | | | | | |
| --- | --- | --- | --- | --- | --- |
| **Residue** | **ΔE_vdW_** | **ΔE_elec_** | **ΔE_polar_** | **ΔE_surf_** | **Total** |
| **A-Y348** | -2.128 | -0.457 | 0.248 | -0.171 | -2.508 |
| **A-Y353** | -0.991 | -0.559 | 0.602 | -0.189 | -1.138 |
| **A-F405** | -3.161 | -0.542 | -0.093 | -0.366 | -4.162 |
| **B-D543** | 0.189 | -46.034 | 42.640 | -0.171 | -3.377 |
| **B-V573** | -2.080 | 1.081 | -0.702 | -0.079 | -1.780 |
| **B-T574** | -1.777 | -5.850 | 5.211 | -0.187 | -2.603 |

**Table S6. Energy decomposition of binding free energy of G to TLR8.** All units are kcal/mol.

| **TLR8-G** Binding free energy: -20.99kcal/mol | | | | | |
| --- | --- | --- | --- | --- | --- |
| **Residue** | **ΔE_vdW_** | **ΔE_elec_** | **ΔE_polar_** | **ΔE_surf_** | **Total** |
| **A-Y348** | -1.175 | -0.188 | -0.083 | -0.236 | -1.682 |
| **A-Y353** | -1.082 | -0.320 | 0.364 | -0.169 | -1.206 |
| **A-F405** | -3.173 | -0.874 | -0.945 | -0.415 | -5.406 |
| **B-D543** | 0.396 | -66.000 | 60.051 | -0.121 | -5.674 |
| **B-D545** | -1.254 | -44.897 | 45.192 | -0.163 | -1.122 |

**Table S7. Details of hydrogen bonds between H and TLR7.**

| **Model** | **Acceptor** | **Donor** | **Occupied (%)** | **Distance (Å)** | **Angle (︒)** |
| --- | --- | --- | --- | --- | --- |
| **TLR7-H** | B-D555@OD2 | H@N1 | 98.75 | 2.77 | 162.33 |
|  | B-D555@OD1 | H@N | 90.80 | 2.82 | 162.83 |
|  | B-T586@O | H@N1 | 34.88 | 2.88 | 147.63 |
|  | H@N2 | B-T586@N | 17.14 | 2.94 | 159.43 |

**Table S8. Details of hydrogen bonds between R and TLR7.**

| **Model** | **Acceptor** | **Donor** | **Occupied (%)** | **Distance (Å)** | **Angle (︒)** |
| --- | --- | --- | --- | --- | --- |
| **TLR7-R** | B-D555@OD2 | R@N1 | 53.17 | 2.77 | 152.95 |
|  | B-D555@OD1 | R@N | 46.73 | 2.83 | 157.16 |
|  | B-T586@O | R@N1 | 43.83 | 2.87 | 151.92 |
|  | B-D555@OD2 | R@N | 36.48 | 2.83 | 154.48 |
|  | B-D555@OD1 | R@N1 | 32.93 | 2.76 | 153.55 |
|  | R@N2 | B-T586@N | 30.08 | 2.93 | 159.73 |
|  | R@O1 | B-T586@OG1 | 17.04 | 2.87 | 159.07 |

**Table S9. Details of hydrogen bonds between G and TLR7.**

| **Model** | **Acceptor** | **Donor** | **Occupied (%)** | **Distance (Å)** | **Angle (︒)** |
| --- | --- | --- | --- | --- | --- |
| **TLR7-G** | B-D555@OD2 | G@N | 99.10 | 2.75 | 164.13 |
|  | B-D555@OD1 | G@N1 | 94.70 | 2.79 | 161.33 |
|  | B-T586@OG1 | G@N4 | 43.88 | 2.85 | 160.22 |
|  | B-T586@O | G@N1 | 12.99 | 2.88 | 145.02 |

**Table S10. Details of hydrogen bonds between H and TLR8.**

| **Model** | **Acceptor** | **Donor** | **Occupied (%)** | **Distance (Å)** | **Angle (︒)** |
| --- | --- | --- | --- | --- | --- |
| **TLR8-H** | B-D543@OD2 | H@N | 52.22 | 2.79 | 160.12 |
|  | B-D543@OD1 | H@N1 | 46.48 | 2.84 | 159.97 |
|  | B-D543@OD1 | H@N | 44.53 | 2.76 | 160.39 |
|  | B-D543@OD2 | H@N1 | 39.28 | 2.83 | 161.78 |
|  | B-T574@OG1 | H@N1 | 30.68 | 2.89 | 152.73 |
|  | B-D545@OD1 | H@O1 | 17.19 | 2.75 | 160.21 |

**Table S11. Details of hydrogen bonds between R and TLR8.**

| **Model** | **Acceptor** | **Donor** | **Occupied (%)** | **Distance (Å)** | **Angle (︒)** |
| --- | --- | --- | --- | --- | --- |
| **TLR8-R** | B-D543@OD2 | R@N | 93.60 | 2.80 | 162.14 |
|  | B-D543@OD1 | R@N1 | 72.51 | 2.86 | 160.31 |
|  | B-T574@O | R@N1 | 25.29 | 2.87 | 142.02 |
|  | R@N2 | B-T574@N | 16.84 | 2.94 | 163.94 |

**Table S12. Details of hydrogen bonds between G and TLR8.**

| **Model** | **Acceptor** | **Donor** | **Occupied (%)** | **Distance (Å)** | **Angle (︒)** |
| --- | --- | --- | --- | --- | --- |
| **TLR8-G** | B-D543@OD1 | G@N | 70.36 | 2.80 | 162.88 |
|  | B-D543@OD2 | G@N1 | 62.17 | 2.83 | 158.31 |
|  | B-D543@OD2 | G@N | 22.74 | 2.79 | 160.08 |
|  | B-D543@OD1 | G@N1 | 16.29 | 2.85 | 158.24 |
|  | B-G572@O | G@N4 | 20.49 | 2.85 | 149.34 |


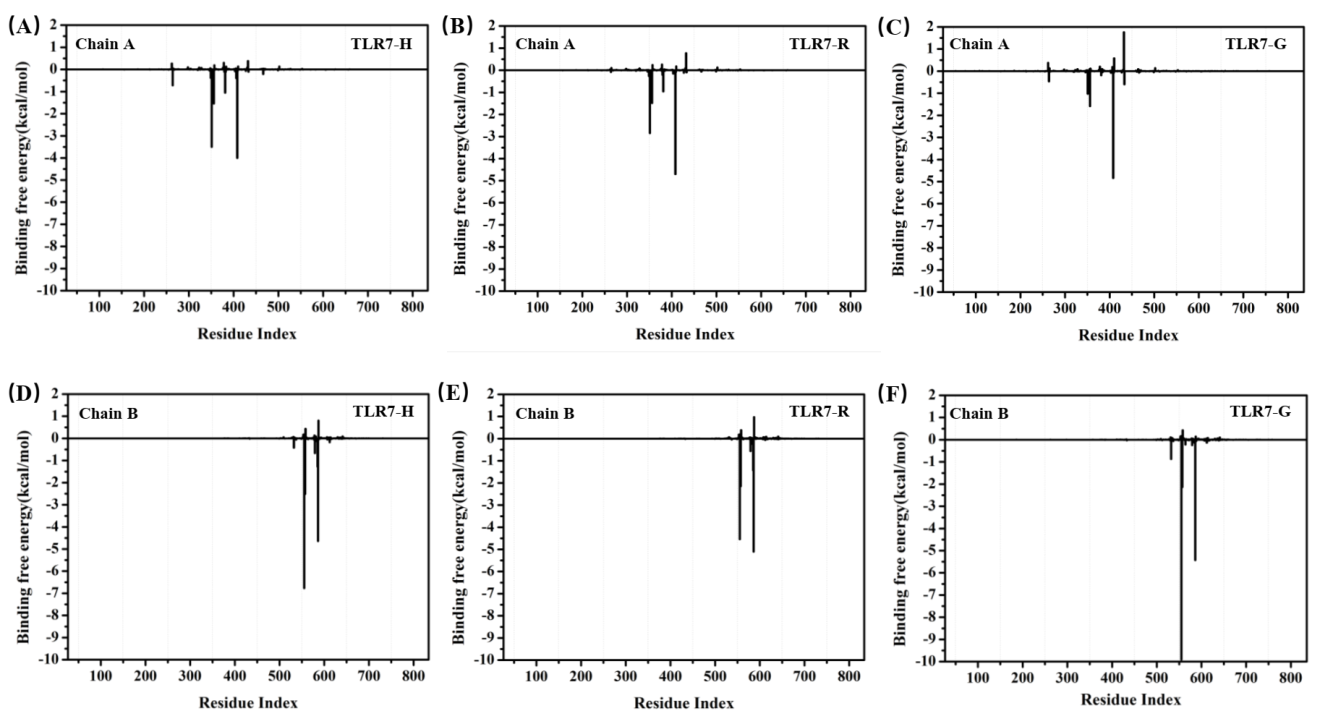


**Fig S1. The binding free energies between each residue of TLR7 and H, R and G.**


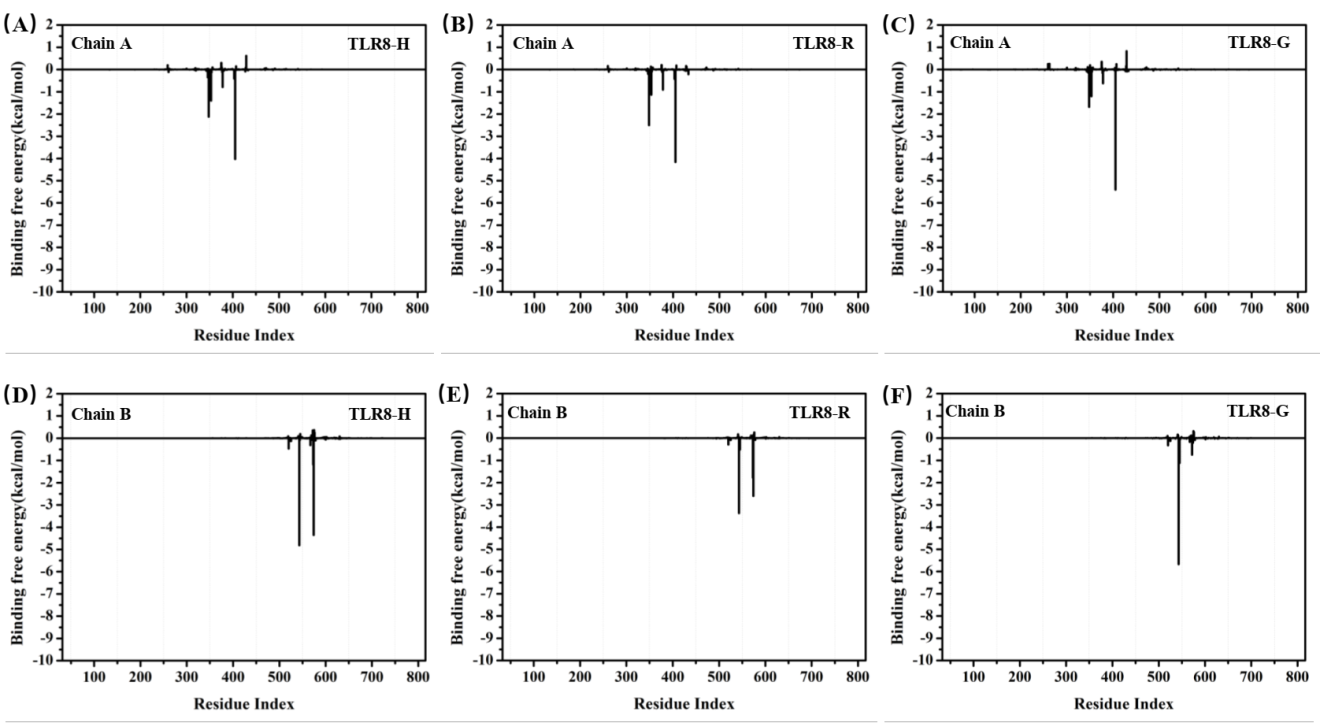


**Fig S2. The binding free energies between each residue of TLR8 and H, R and G.**
