# Supplementary material for: Molecular dynamics simulations reveal the selectivity mechanism of structurally similar agonists to TLR7 and TLR8
Source: PLoS One. 2022 Apr 22;17(4):e0260565. doi: 10.1371/journal.pone.0260565 (PMC9032342; doi:10.1371/journal.pone.0260565)
Supplement: Attachment — Submitted filename: Response to Reviewers.docx [file pone.0260565.s003.docx]

**Responses to reviewer#1**

The authors have addressed all the comments, however, there are additional comments that need to be addressed by authors before the acceptance of the manuscript.

Many thanks for taking your time to review our responses. We have incorporated all the suggestions of the reviewer that helped to improve this manuscript, and highlighted the revisions in red.

1. Page 4, line 77. Kindly remove this part from the paragraph, "In the late 70s, the first case of MD is Bovine pancreatic trypsin inhibitor [26]. Especially in recent years, benefit from the development of modern hardware and force fields, MD simulation has made huge progress. Protein conformation dynamics that is hardly available by current experimental techniques can be observed using MD simulations [27-34]." As it is extra and does not add scientific value to your manuscript.

So the updated paragraph will be "Molecular dynamics (MD) simulation is a very established computational technique to understand the protein structure-function relationship and guide the drug design. MD simulation has played an important role in characterizing receptor-ligand interaction .... with a simulation time of 10-100us [55-57].

**Response:** Many thanks for the constructive suggestion. This paragraph has been reduced. The revised paragraph in section 1 is following:

Molecular dynamics (MD) simulation is a very established computational technique to understand the protein structure-function relationship and guide the drug design. MD simulation also has played an important role in characterizing receptor-ligand interaction [26-28], providing the guidance of structure-based drug design [29-31] and typical conformations for virtual screening [32-37]. Besides, it helps to reveal the novel binding sites which have not been captured by NMR and X-ray crystallographic analysis, for example, cryptic binding sites in HIV-1 integrase [38-41]. Up to date, MD simulations have been successfully applied to many large systems, such as the complete HIV1 capsid with 64 million atoms up to 100ns [42-45]. MD simulation has been also instrumental on understanding the protein folding and function regulation with a simulation time of 10-100us [46-48].

2. Page 11, line 246. "In another trajectory analysis, we also examine the conformational changes of agonists. The heavy atom RMSD of agonists was calculated in the six TLR-agonist systems (Figs 5B and 5E) with respect to the initial conformation."

Kindly change it to " We also examine the conformational changes of agonists. The heavy atoms' RMSD of agonists was calculated in the six TLR-agonist systems (Figs 5B and 5E) with respect to the initial conformation."

**Response:** We are thankful to the reviewer’s positive suggestion. The revised paragraph in section 3.2 is following:

We also examined the conformational changes of agonists. The heavy atoms' RMSD of agonists was calculated in the six TLR-agonist systems (Figs 5B and 5E) with respect to the initial conformation.

3. Page 16, line 349. "The occupancy of hydrogen bond between N1 and T574 of TLR8-G is zero."

Kindly remove this sentence since there is no hydrogen bond between N1 and T574 of TLR8-G.

**Response:** We are thankful to the reviewer’s valuable suggestions. This sentence has been removed.
